# Cluster-randomized Studies in Educational Research: Principles and Methodological Aspects

**DOI:** 10.3205/zma001103

**Published:** 2017-05-15

**Authors:** Jens Dreyhaupt, Benjamin Mayer, Oliver Keis, Wolfgang Öchsner, Rainer Muche

**Affiliations:** 1 Ulm University, Institute of Epidemiology and Medical Biometry, Ulm, Germany; 2 Ulm University, Office of the Dean of Studies for Medicine, Ulm, Germany; 3 University Hospital Ulm, Department of Cardiac Anesthesiology, Ulm, Germany

**Keywords:** cluster randomization, structural equivalence, educational research, study, sample size calculation, statistical analysis

## Abstract

An increasing number of studies are being performed in educational research to evaluate new teaching methods and approaches. These studies could be performed more efficiently and deliver more convincing results if they more strictly applied and complied with recognized standards of scientific studies. Such an approach could substantially increase the quality in particular of prospective, two-arm (intervention) studies that aim to compare two different teaching methods. A key standard in such studies is randomization, which can minimize systematic bias in study findings; such bias may result if the two study arms are not structurally equivalent. If possible, educational research studies should also achieve this standard, although this is not yet generally the case. Some difficulties and concerns exist, particularly regarding organizational and methodological aspects. An important point to consider in educational research studies is that usually individuals cannot be randomized, because of the teaching situation, and instead whole groups have to be randomized (so-called “cluster randomization”). Compared with studies with individual randomization, studies with cluster randomization normally require (significantly) larger sample sizes and more complex methods for calculating sample size. Furthermore, cluster-randomized studies require more complex methods for statistical analysis. The consequence of the above is that a competent expert with respective special knowledge needs to be involved in all phases of cluster-randomized studies.

Studies to evaluate new teaching methods need to make greater use of randomization in order to achieve scientifically convincing results. Therefore, in this article we describe the general principles of cluster randomization and how to implement these principles, and we also outline practical aspects of using cluster randomization in prospective, two-arm comparative educational research studies.

## Zusammenfassung

In der Lehrforschung werden immer häufiger Studien zur Evaluation neuer Lehrmethoden und Ansätze durchgeführt, wobei diese Studien bei einer strengeren Anwendung und Einhaltung von anerkannten Standards und Methoden wissenschaftlicher Untersuchungen noch effizienter durchgeführt werden und überzeugendere Ergebnisse liefern könnten. Insbesondere bei prospektiven zweiarmigen (Interventions)-Studien, in denen zwei verschiedene Lehrmethoden verglichen werden sollen, könnte eine entsprechende Vorgehensweise zu einer substanziellen Qualitätssteigerung führen. Ein wesentlicher Standard ist dabei die Randomisierung, mit der systematische Verzerrungen der Studienergebnisse durch Strukturungleichheiten in den zu vergleichenden Studienarmen weitestgehend ausgeschlossen werden können. Dieser Standard sollte möglichst auch bei Studien in der Lehrforschung erreicht werden, wo er sich allerdings aktuell noch nicht allgemein durchgesetzt hat. Es gibt hierbei einige Schwierigkeiten und Vorbehalte, vor allem organisatorische und methodische Aspekte. Insbesondere muss beachtet werden, dass bei Studien in der Lehrforschung bedingt durch die Lehrsituation meist keine individuelle Randomisierung sondern eine Randomisierung ganzer Gruppen (sogenannte Cluster-Randomisierung) vorgenommen werden muss. Im Vergleich zu individuell randomisierten Studien sind bei cluster-randomisierten Studien meist (deutlich) höhere Fallzahlen sowie eine komplexere Methodik der Fallzahlplanung notwendig. Weiterhin erfordern cluster-randomisierte Studien umfassendere Methoden zur statistischen Auswertung. Dies hat zur Konsequenz, dass die praktische Anwendung cluster-randomisierter Studien in allen ihren Phasen der Unterstützung durch einen kompetenten Experten mit entsprechenden Spezialkenntnissen bedarf.

Eine verstärkte Anwendung der Randomisierung in Studien zur Beurteilung neuer Methoden in der Lehre ist notwendig, um wissenschaftlich überzeugende Ergebnisse zu erzielen. Um dazu beizutragen, werden in diesem Beitrag allgemeine Grundlagen der Cluster-Randomisierung beschrieben, deren Umsetzung und praktische Aspekte der Durchführung im Kontext von prospektiven zweiarmigen vergleichenden Studien in der Lehrforschung erläutert.

## 1. Introduction

An increasing number of studies are being performed to evaluate new teaching methods and approaches in educational research, particularly in the field of medicine [1], [2], [3], [4]; the increase has been particularly noticeable in Germany in recent years [5]. These studies should also comply with recognized standards and methods of scientific research. The methods of clinical studies in drug development are well developed [6], [http://www.ich.org/products/guidelines/efficacy/article/efficacy-guidelines.html], and the procedure is standardized worldwide to ensure that meaningful study results are achieved. These standards should therefore be established and applied as far as possible also in educational research studies. In addition to being important for observational studies, these standards are relevant mainly for prospective two-arm (intervention) studies that aim to compare two different teaching methods.

Studies in clinical research require a control arm that is statistically comparable to the test arm (structural equivalence) in order to prove the efficacy or superiority of a treatment. Randomization ensures that all confounders – known and unknown – are distributed equally across the control and test arms and that only random differences, if any, are present at the start of treatment [7]. In the context of clinical studies, randomization does not mean that the patient is simply assigned to a treatment without any obvious criterion, but that a random experiment is formally performed that is independent of the participating clinician. If any other procedure is used to allocate volunteers or patients to the control and test arms, systematic distortions of the results are likely. Randomization is also an accepted method for educational research studies [8].

Educational research studies have some features and special requirements that are relevant for randomization. One of these features is that it is often not meaningful or possible to randomize individual students, for example because students are not always available as a result of their individual semester schedules. Furthermore, studies are usually performed in the natural learning situation of teaching in groups, such as joint lectures or seminars supervised by a lecturer. As a consequence of the above, in educational research studies it makes sense and is necessary to randomize whole groups (cluster randomization) rather than individual students.

In order to obtain scientifically convincing results in the sense of “evidence-based didactics,” educational research needs to make more use of randomization in studies to evaluate new teaching methods and approaches. To help increase the use of randomization, this paper describes the principles of cluster randomization and explains practical aspects in order to facilitate its use in prospective, two-arm comparative studies in educational research.

The first section presents the rationale of randomization. Subsequently, the distinctive features of scientific studies in educational research are discussed. The third section first describes the principles of cluster randomization and then discusses sample size calculation and approaches to analysing cluster-randomized studies, both of which differ from those of studies with individual randomization. Finally, a sample design for an educational research study with cluster randomization is presented as an example of its application.

## 2. The reason for randomization: structural equivalence

In order to show why randomization is necessary, we will first explain some concepts that are relevant to educational research studies.

**Necessity of a control arm:** In educational research, if a new method is used in a study with only one study arm (test arm), one cannot rule out that an observed effect could have arisen also without the new method. Thus, not all observed results in the test arm can be attributed to the new method. The efficacy of a new method only becomes evident if the specific result is better with this method than without it [9]. Therefore, to obtain proof of efficacy a control arm, in which the previously used method is applied, is essential.

**Statistical comparability:** Ensuring that differences in results observed when comparing the control and test arms really can be attributed only to the new method requires statistical comparability of the following:

1. the structure of the control and test arms (structural equivalence);
2. the interactions with students, with the exception of the specific new method being evaluated (equivalence of treatment conditions); and
3. the observations (equivalence of observations).

In this context, “statistically comparable” means that the control and test arms differ as little as possible and at the most by chance.

**Structural equivalence:** Structural equivalence is given when the composition of the test and control arms is statistically comparable with respect to potential so-called “confounders” [10]. Examples of such confounders are age and gender, which often influence results. In educational research, additional factors are personality factors, such as educational background and special skills, as well as interests and activities. Randomization is one approach to achieve structural equivalence. Random allocation of students to the control or test arm allows one to achieve a similar distribution of known and unknown confounders across both arms, or at least to assume that it has been achieved. In addition, stratification can be used to balance (a few) important known confounders between the control and test arms (see Section 4.2); however, the feasibility of stratification is limited in educational research.

**Equivalence of treatment conditions and observations**: Equivalence of treatment conditions is achieved if all students in all groups receive and experience the same treatment conditions, apart from the new method being assessed. These treatment conditions (e.g. the same time for seminars, the same conditions for written examinations) should be specified in as much detail as possible in a study protocol – which should be written also for educational research studies – so that when the results are interpreted it is clear “what” was compared and under what conditions the observed effect arose. The control and test arms are considered to have “equivalence of observations” when situations are always observed and assessed according to the same rules (standardized conditions), e.g. the same evaluators are used to grade examinations. In clinical research, one procedure to achieve equivalence of treatment conditions and observations is the masking (blinding) of participants and investigators as to the treatment being received. In educational research studies, however, blinding is usually not possible and is only conceivable for evaluations, e.g. results in problem-based learning (PBL) can be evaluated by independent people who are not involved in the study.

If an educational research study has the above mentioned characteristics and if the success in the test arm is that much greater than in the control arm that the difference cannot be a coincidence, the study has shown that only the new method can be responsible for the outcome. However, if the control and test arms differ in other respects (i.e. they do not have structural equivalence or equivalence of treatment conditions or observations), the findings are difficult to interpret because an observed effect can no longer be exclusively attributed to the new method (blended effects). In such a situation, the specific effect of the new teaching method cannot be calculated and the study results usually cannot be interpreted according to the study question. Consequently, randomization is an important instrument to achieve structural equivalence in studies of the efficacy of new methods in educational research.

## 3. Conditions and requirements of educational research studies

Educational research studies take place in a special context. They usually face a natural cluster structure, because teaching is usually performed in groups of students. Examples are seminar groups, PBL groups, and groups of students attending a joint lecture. The group size varies greatly and ranges from 5-8 (PBL groups) to over 100 students in a joint lecture. The size of seminars varies, whereas in Germany one can assume because of legal specifications that in medicine a seminar group has up to 20 students [http://www.gesetze-im-internet.de/_appro_2002/index.html]. All students within a group are exposed to the same conditions, such as the same teacher or the same facilities and times. Consequently, the intervention being studied (e.g. the use of a new teaching method by the lecturer) takes place at the level of the whole group. Thus, the outcomes of students in the same group are usually more similar than those of students in different groups. Furthermore, in addition to students’ individual semester schedules an additional aspect to be considered in educational research studies is the time availability of appropriate resources (such as lecturers, seminar rooms, laboratories, lecture halls, computer pools). Also, if the number of students is limited and the group size is predefined, then a limited number of possible groups is available for a particular study. The above mentioned aspects mean that individual randomization can hardly be used in educational research studies, but that cluster randomization is meaningful and feasible. In cluster randomization, student groups or lecturers (who supervise groups of students) are randomized to the test and control arms, whereby the special characteristics mentioned above have to be taken into account.

Various outcome variables are conceivable in educational research studies:

- Evaluation results (e.g. student satisfaction, self-assessment of the achieved competence)
- Results of course assessments/examinations (level of competence achieved)
- Measurement of the necessary learning effort (e.g. tracking of study time)
- Accompanying effects of studying the learning material (e.g. motivation curves, enthusiasm for the topic, awakening of interest, career planning)

In the following, we consider metric outcome variables (e.g. examination scores), which are assumed to be approximately normally distributed. We do not cover binary outcome variables (e.g. passed: yes/no) in this paper because studies examining such variables usually require a significantly larger sample size and are thus almost impossible to conduct in the field of educational research.

## 4. Cluster randomization and its use in educational research

The following sections explain the principles of cluster randomization and how they can be applied in the field of educational research. Furthermore, they provide information about study implementation, sample size calculation, and statistical analysis.

### 4.1. Definition and motivation 

**Figure 1:**
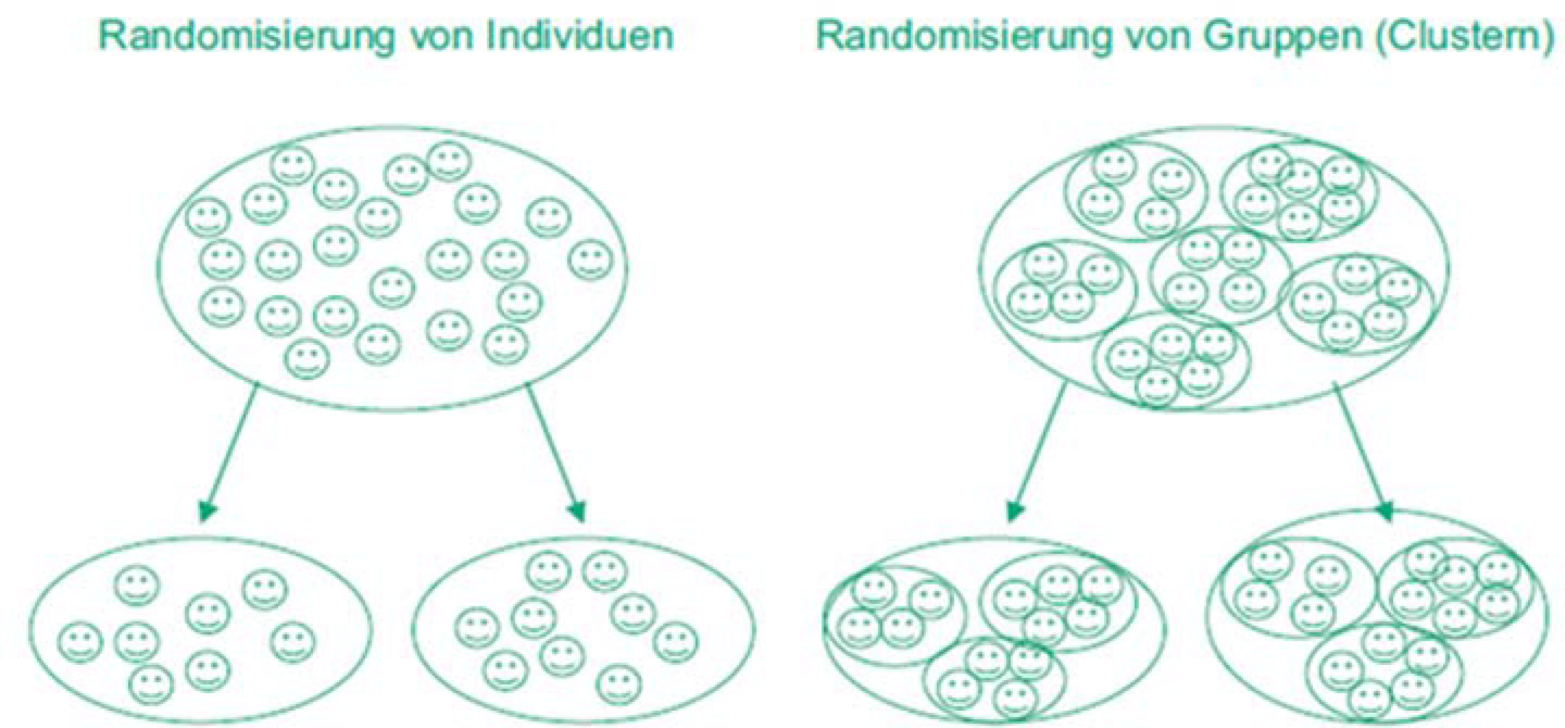
Randomization of individuals vs. randomization of clusters (reproduced from [15]).

In a cluster-randomized study (also called a “group-randomized study,” “community randomization study,” or “community intervention study”), entire social groups or clusters of individuals are randomized, rather than individuals (see Figure 1 (Fig. 1)). 

To date, this type of study has been performed particularly to evaluate non-therapeutic interventions, such as training programs, prevention programs, and health promotion measures. For the reasons mentioned in the last section, it seems to make sense to use cluster-randomized studies also in the field of educational research. Published cluster-randomized studies include studies with both small and large clusters: clusters are defined frequently by households, families, neighborhoods, municipalities, school classes, employers, hospitals, and doctors’ practices. Thus, the number of individuals per cluster can range from 2 to several thousands. Various cluster sizes are also conceivable in educational research (see Section 3).

The main motivation for conducting a cluster-randomized study is wanting to avoid or reduce a contamination bias. If individual randomization was used in an educational research study, such a bias (distortion or systematic error) could be caused by interactions between individuals in different study arms. For example, if students in the control arm were individually randomized they could easily be encouraged by students in the test arm to carry out the specific methods of the test arm whose efficacy is being tested. Cluster randomization does not entirely eliminate this risk, however, but only reduces it. The widespread use of social media, such as Facebook, plays a role in contamination bias in educational research studies. Another important reason for the use of cluster randomization in educational research is the existence of natural clusters, because learning is usually done in groups (see Section 3).

Table 1 (Tab. 1) presents important advantages and disadvantages of cluster randomization in the context of educational research. 

#### 4.2. Designs

In cluster-randomized studies, a distinction can be drawn between a completely randomized design, a stratified randomized design, and a matched design. In a completely randomized design, the clusters are assigned randomly to the groups and are neither stratified nor matched. An example is an educational research study in which seminar groups are randomized into either the test arm or the control arm. In the stratified randomized design, randomization is stratified on the basis of (a few) important known confounders so that the distribution of the confounders is similar in the test and control arms. Stratification is performed according to factors that are strongly associated with the outcome variables, such as cluster size, gender, or day. An example is an educational research study in which the day on which a seminar takes place is assumed to influence the outcome variable (i.e. it is a confounder). In this case, stratification can first be performed according to the day (e.g. Monday/Wednesday/Friday) and then seminar groups can be randomized into either the test or the control arm within each day. In this way, the confounder “day” is distributed almost equally across both arms. In the matched design, pairs of clusters are formed that are as similar as possible with respect to important factors that affect the outcome variable. One cluster of the pair is randomized to the test arm and the other to the control arm. This represents a good way to balance confounders (e.g. characteristics from the baseline evaluation, such as gender, semester, previous grade) between the two arms and thus to make the arms comparable. Not too many criteria should be used for the matching, however, because it may then become impossible to find a cluster that can form a pair with another.

As a result of the conditions and requirements described in Section 3 (specifications for cluster size; limited number of students and consequently limited number of clusters; availability of resources; individual semester schedules), educational research studies often can be assumed to have a relatively small number of clusters with a more or less fixed cluster size. In this context, stratified and matched designs are probably only feasible under special conditions. An example would be a multicenter study conducted at different institutions. For this reason, the completely randomized design will prevail in educational research.

#### 4.3. Practical implementation

Educational research studies usually have to be reported to the responsible ethics committee. However, often it is not necessary to obtain written informed consent from the participating students and sufficient just to inform them about the study [11].

Inclusion and exclusion criteria must be defined at both the individual level (students) and the cluster level (teachers). One problem is that blinding usually is not possible in educational research studies. Consequently, there is a risk of bias in the outcome variable. This risk should be countered by measures to achieve equivalence of treatment conditions and observations. Examples are a strong standardization of the general approach and perhaps a blinded assessment of the outcome, e.g. by a third evaluator who is not involved in the study and who has no knowledge of the respective student's assignment to the test or control arm.

#### 4.4. Sample size calculation

##### 4.4.1. Why is sample size calculation different?

Cluster randomization generates a special data structure, whereby observations within the clusters usually are more similar than observations from different clusters (i.e. there is statistical dependence). In the context of educational research studies, this means that the results of students within the same seminar group (e.g. grades in the written exam) are more similar than the results of students in different seminar groups. This results in a loss of efficiency and power, which affects sample size calculation: the effective sample size of a cluster-randomized study (i.e. the number of truly statistically independent individual observations) is lower than the actual sample size (i.e. the number of recruited students). Therefore, standard procedures that assume the statistical independence of all observations are unsuitable for calculating sample size for cluster-randomized studies and evaluating data from these studies. The use of standard procedures to calculate sample size would lead to studies with too little power in which the chance of proving a difference between the study arms that is actually present would be (significantly) lower than assumed in the calculation. In educational research, this may result in a new teaching method that is actually better not being recognized as such by the study, for example.

##### 4.4.2. Determination of similarity - the intracluster correlation coefficient (ICC)

The intracluster correlation coefficient (synonym: intraclass correlation coefficient, ICC; ρ) is used to quantify the similarity of observations within a cluster compared with observations from different clusters. The ICC can be defined in various ways [12]; for metric outcome variables, it is often defined as a quotient of variances [13], [14]:


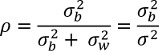


whereby 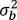 is the variance between the clusters, 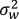 the variance within the same cluster, and 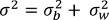 the total variance. This definition allows the ICC to be interpreted as the share of the total variance accounted for by the variance between the clusters, assuming that the variance 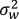 is constant in each cluster. With this definition, the ICC can take values between 0 and 1. Its size is a measure of the strength of the similarity of the observations within the cluster compared with the similarity of the observations between the clusters. If the ICC is 1, the observations within each cluster are the same. In the context of educational research studies this would mean, for example, that in each seminar group all the students have the same examination grade (but not necessarily that all seminar groups in the study have the same grade). The ICC has the value 0 if all observations are statistically independent. In the case of educational research studies, this would mean, for example, that the students’ examination grades within the same seminar group are not dependent, i.e. the seminar group has no influence on the examination grades.

Estimating the ICC a priori is often a challenge. The ICC can be calculated from data from a pilot study or from the literature, for example. Therefore, publications of cluster-randomized studies should include the post hoc calculation of their ICC, so that it is available for similar studies [15], [16]. Furthermore, the ICC is only an estimate from a sample and thus subject to uncertainty (confidence interval [17]). This is of particular importance for educational research studies because often only small studies with few clusters can be performed, so that the ICC cannot be reliably estimated.

In addition, different calculation methods can influence the value of the ICC. An overview of ICC calculation methods suitable for metric outcome variables is given in [18]. For binary outcome variables, corresponding methods are available in [19] and [20].

##### 4.4.3. The design effect (DE)

In order to achieve the same power in a cluster-randomized study as in a study with individual randomization, usually more individuals have to be recruited in the former. The sample size required for a cluster-randomized study is calculated by multiplying the sample size of a study with individual randomization with the design effect (DE), which is calculated from the ICC ρ and the fixed cluster size m:

**DE = 1+ρ·(m-1)**

The result is a total sample size and subsequently a number of clusters (with fixed cluster size) for a given power. For educational research studies, this means that initially a total number of students is calculated and then, on the basis of this, the number of seminar groups (with fixed group size m).

If cluster sizes are unequal, m can be replaced by the arithmetic mean or by the maximum cluster size. The use of the arithmetic mean of the cluster size is useful when there is little variability in cluster size [12]; the use of the maximum cluster size is a conservative approach. If the ICC is ρ=0 (statistical independence of the observations, see above), DE=1, which means that the cluster-randomized study has the same sample size as a corresponding study with individual randomization. In this case, the formation of clusters has no influence on the sample size. In practice, most ICCs are between 0.00 and 0.20, although there is a very wide range [21].

##### 4.4.4. Procedure for calculating sample size

**Figure 2:**
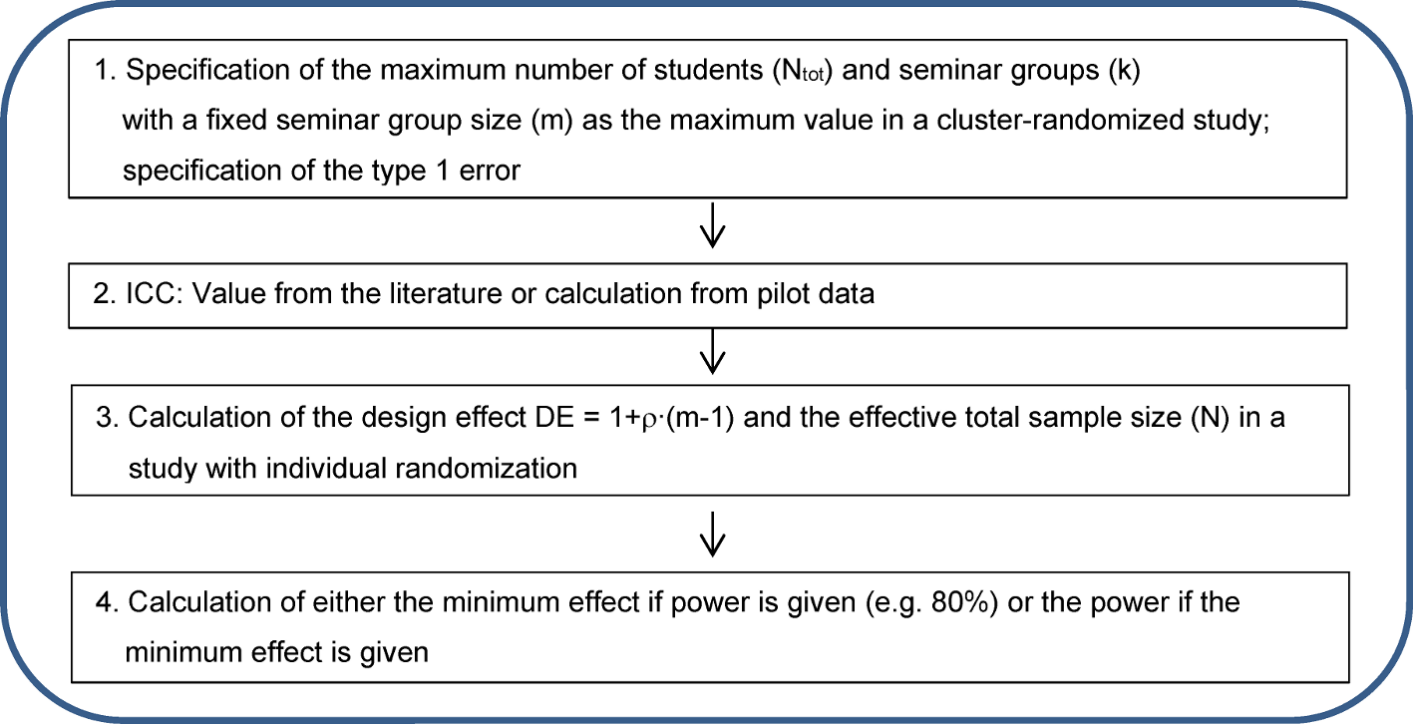
Schema for calculating power or minimum effect in educational research studies if the sample size is predetermined

In general, two approaches can be considered when planning a study. One is an exploratory approach in which a minimum effect can be calculated for the given maximum sample size with a given power and cluster size or a power can be calculated for a given minimum effect and cluster size [22]. This is particularly useful when only a limited number of observations are available. Figure 2 (Fig. 2) shows the schema for calculating the power or minimum effect for a given sample size in educational research studies.

**Figure 3:**
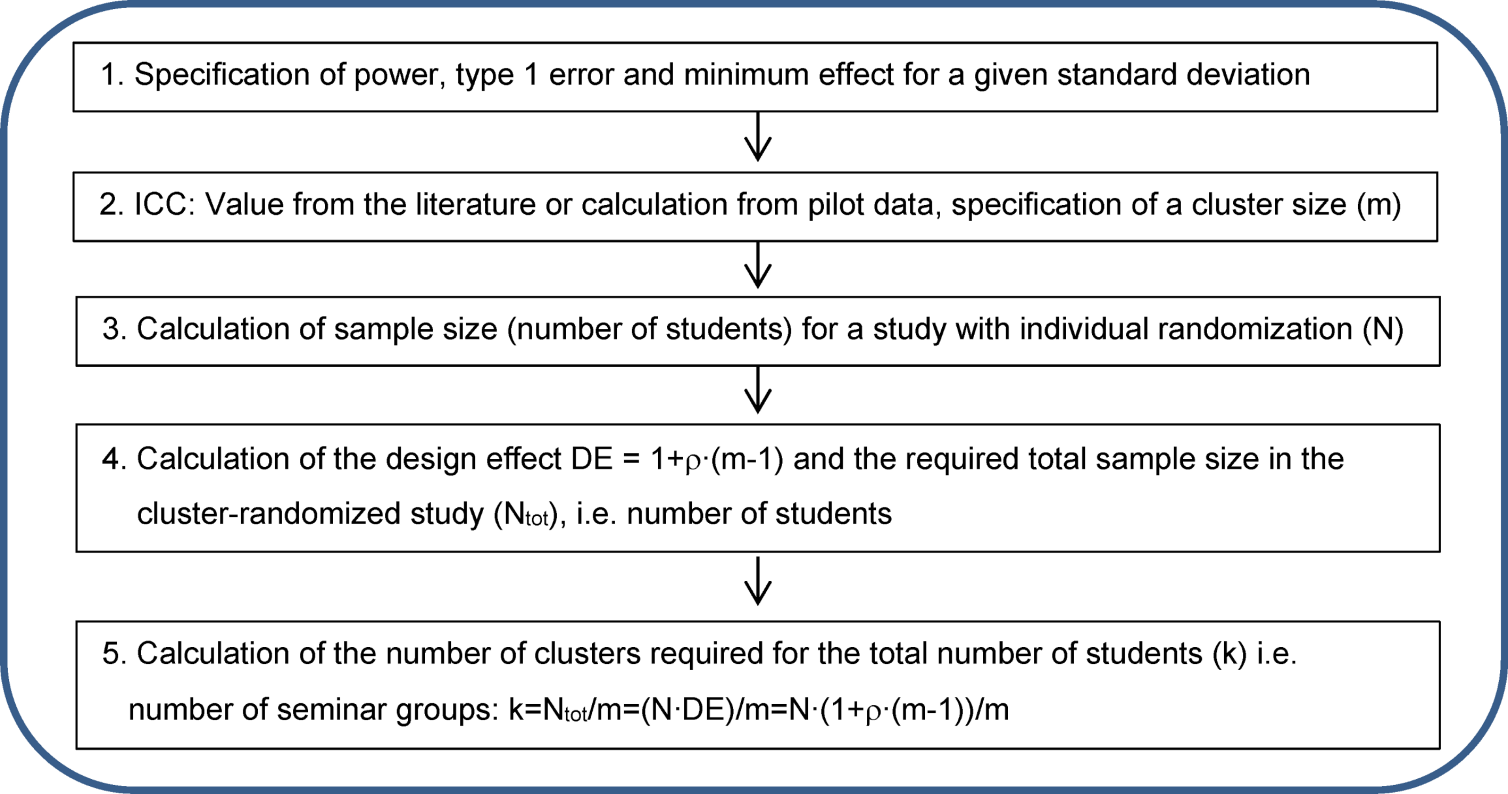
Schema for calculating sample size in educational research studies with a given power and minimum effect

The other is a confirmatory approach: a sample size (i.e. the number of students and a resulting cluster number) is calculated for a given power and a predetermined minimum effect. Figure 3 (Fig. 3) shows the schema for calculating sample size in educational research studies with a given power and minimum effect. 

However, because of the special conditions in educational research (limited number of students and therefore of clusters and a given cluster size, see above and Section 3) confirmatory studies often cannot be implemented.

If additional covariates are to be included in the planning of a cluster-randomized study, the definition of the ICC can be expanded according to [14]. Simulation is another option, especially for complex study designs (e.g. if several covariates are to be considered in a longitudinal design, presence of additional hierarchical levels) (e.g. [23], [24], [25], [26]).

#### 4.5. Statistical Analysis

In order to take into account the statistical dependencies within the clusters (in educational research studies, an example would be the dependence of examination results of students in the same seminar group), a cluster adjustment has to be performed during the analysis [12]. A so-called “naive analysis” (cluster adjustment is not performed; standard methods are applied, such as a two-sample t-test) can result in the estimated confidence intervals and p values being too small [27], [28]. In educational research studies, this would result in studies being falsely reported as significant and thus new teaching methods appearing to be better than they actually are.

The methods used to plan a study should also be used to analyze it [23], [28], whereby the methods depend on the study design (see above). In the statistical analysis, a distinction can be made between the analysis at the cluster level and at the individual level [28], [13]. Because of the complexity of the statistical methods, the support of a competent expert (e.g. statisticians with appropriate special knowledge) is recommended in particular for the analysis. Almost all medical faculties in Germany that have a medical or dental school are connected with methodically versed institutes (such as biometry departments), which could provide expertise accordingly.

Cluster-level analysis is the simplest evaluation method for a cluster-randomized study and can be viewed as a two-step process: initially, a composite measure (cluster-level summary) is calculated for each cluster (first stage), and then the composite measures are compared with a suitable statistical test (second stage), see e.g. [16]. In educational research studies, for example, the mean cluster values (e.g. the mean grade in each seminar group) can be used in the analysis (e.g. ordinary two-sample t-test) instead of the students’ individual results. Covariates can be considered in a simplified way via regressions [13]. Analysis at the cluster level is robust, especially if the number of clusters is small, but has the disadvantage that it does not take into account the variability within the clusters. An alternative is to adjust univariate test statistics (e.g. the t value in the t-test) by considering the design effect, whereby the individual results may be evaluated as being statistically independent [15], [29].

Individual-level analysis is an alternative approach that is especially relevant for strongly varying cluster sizes, a situation in which cluster-level analysis is less efficient. The adjusted two-sample t-test is one simple statistical procedure that also allows an analysis to be performed at the individual level [28]. If additional covariates are to be considered, regression models with random effects, mixed effects regression models, or generalized estimating equations (GEE models) can be applied. These approaches also allow factors to be considered as potential influencing variables in the event that stratification was not possible during cluster randomization, even though known prognostic factors were present. The use of one of these approaches has advantages over using cluster-level analysis methods because the effects of covariates can be examined on the same level as the effect of the study arm (as a regression coefficient with a confidence interval and p value). Individual-level analysis methods have the disadvantage, however, that they are less robust when there is a small number of clusters. One recommendation, therefore, is to use cluster-level analysis methods if there are fewer than 15 to 20 clusters per study arm [13]. In studies with a larger number of clusters, individual-level analysis methods can have advantages, especially if cluster size is highly variable.

#### 4.6. Reporting

The CONSORT Statement was developed for the reporting of randomized clinical trials but has been extended to cluster-randomized studies by Campbell et al. [30]. The extended CONSORT statement considers the special characteristics of a cluster-randomized study and should be considered when publishing such a study. The stipulations include the following:

- Describe the reasons for using cluster randomization
- Name the unit of randomization and the intervention
- In addition to the number of individuals, state the number of clusters and their size
- Show structural equivalence not only at the individual level but also at the cluster level
- Calculate and report the ICC (see above)
- Analyze the drop-outs on both the individual and the cluster level
- Draw a flowchart to show the number of study participants and clusters in the course of the study

## 5. Example of application

**Figure 4:**
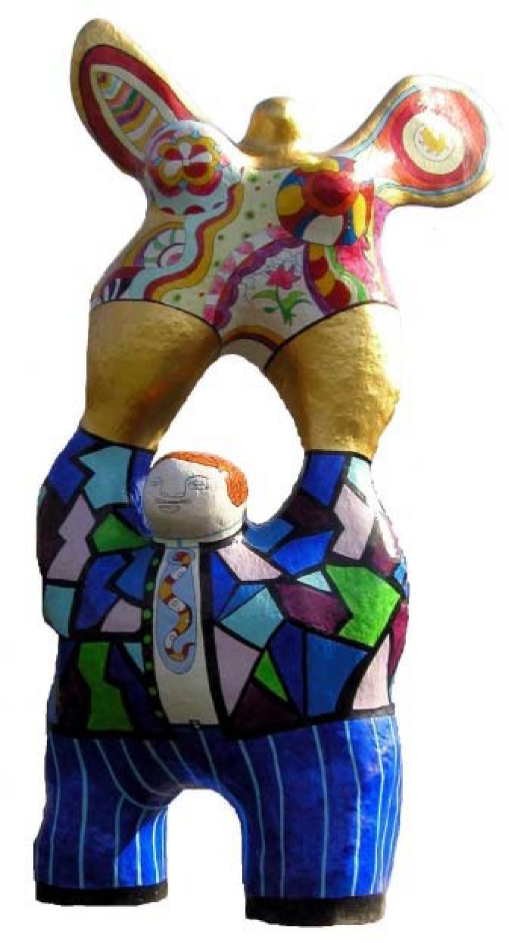
NANA in front of Ulm University

In this section, we will use an example to outline the planning, implementation, and analysis of a cluster-randomized study in educational research. The example is based on the NANA study [31], which is used to illustrate studies in clinical research. The study is conducted as a two-arm, prospective observational study and compares people with a sweet tooth (“NAschkatzen” in German) with people who like to nibble (“NAgetiere” in German) with regard to parameters such as the body mass index. The name of the study also is of relevance to Ulm University, which has a large NANA statue in front of it (see Figure 4 (Fig. 4)).

The example cluster-randomized study is supposed to evaluate whether the use of a new “active seminar concept” (as part of the NANA study) influences the test results in the biometry education of medical students. The “active seminar concept” (planning, implementation, and analysis of a small empirical study during the seminar) is to be compared with the previous standard concept (working on practice exercises in the form of a “classical seminar”). For the study (balanced, prospective, cluster-randomized), whole seminar groups are to be randomized to either a test arm (“active seminar concept”) or a control arm (“classical seminar”). The study is to be carried out at the medical faculty of Ulm University during a winter semester.

A maximum total of about 320 students can be assumed. The students are supervised in seminar groups, each with one lecturer and approximately 20 students. This results in a maximum number of 16 clusters (i.e. seminar groups) in the overall study (i.e. 8 clusters per study arm), making it a smaller study [32].

**Figure 5:**
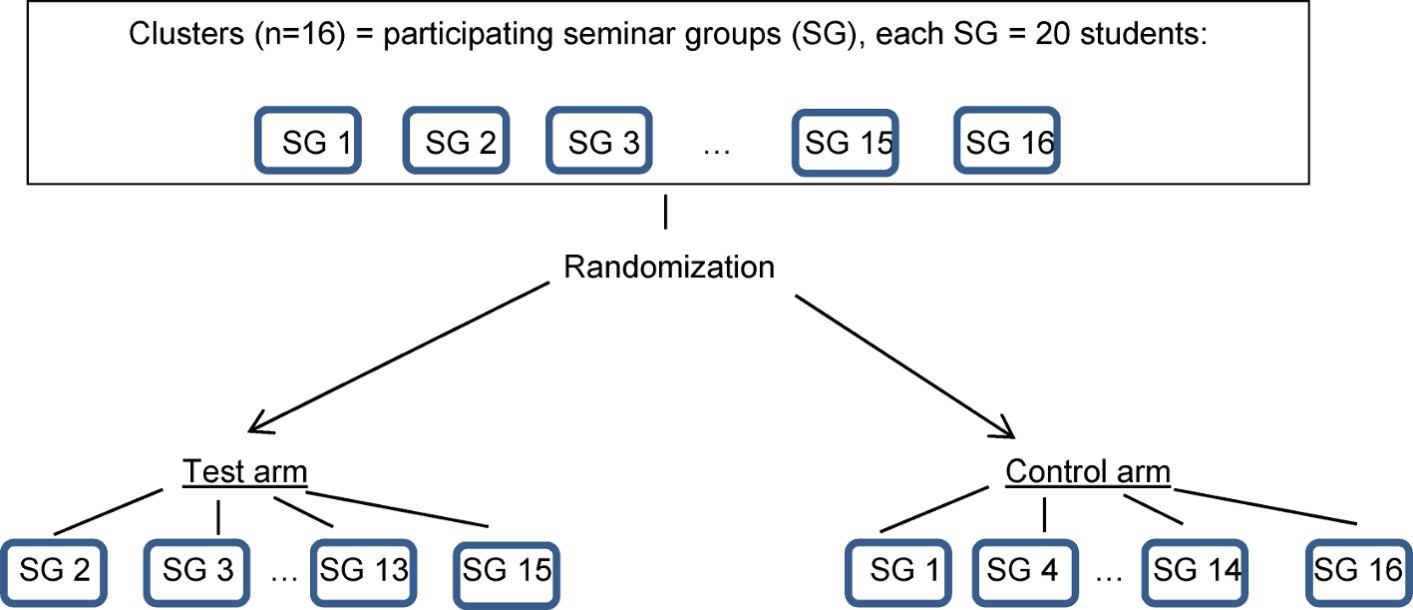
Sample result of cluster randomization for the sample study

Figure 5 (Fig. 5) shows a possible result of the cluster randomization for the example study.

The primary outcome variable is the number of points achieved in the examination, assessed for the individual students (i.e. on the individual level). Because the composition of the groups plays a role, in addition to the influence of the lecturer, we can assume that the examination results of the students in the same seminar group are more similar than the results of students in different seminar groups. The outcome variable is assumed to be metric and approximately normally distributed.

**Pilot data:** The results of the cohort from the winter semester 2015/2016 are available as pilot data: an arithmetic mean of 92.5 points (maximum score: 120) was determined with a standard deviation of 9.16 points (see Table 2 (Tab. 2)).

The results of the pilot data are used for the control arm. For the test arm, the score is assumed to improve by a mean of 3 points (i.e. from 92.5 to 95.5). The ICC for the outcome variable “points” was estimated by a linear mixed effects regression model [12]: the model fit resulted in the variances 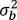 = 1.67 and 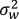 = 82.36, so that the ICC is estimated to be ρ=1.67/(1.67+82.63)=0.02. The design effect (DE=1+ρ·(m-1)) is thus calculated as DE=1+0.02·(20-1)=1.38, whereby m represents the mean cluster size (here: number of students per seminar group; m=20). 

**Study planning: **Both of the approaches mentioned in Section 4.4 (exploratory and confirmatory approaches) will be applied below to calculate the sample size for the example study.

Exploratory approach: Calculation of the power or minimum effect for a particular sample size

The implementation of steps 1 to 4 of the schema shown in Figure 2 (Fig. 2) is described below for the example study. Assuming a maximum number of 320 students per semester, a maximum of 16 clusters (seminar groups with 20 students each) are possible in the planned study. For a design effect of 1.38 (calculation: see above), the maximum number of 320 students in the cluster-randomized study corresponds to an effective sample size of a maximum of approximately 320/1.38 ~ 232 students (even number because of 1: 1 randomization) in a study with individual randomization (i.e. 116 students per study arm). For this sample size, in order to achieve a power of 80% with a two-sided type 1 error of 5% the two-sample t-test requires a minimum difference of 3.4 points (with a standard deviation of 9.16), which corresponds with an effect size of 0.37 according to Cohen (small effect). If a difference of 3 points is assumed (original planning), a two-sided type 1 error of 5% will achieve a power of only 70% (with a given standard deviation of 9.16). Table 3 (Tab. 3) shows the impact of different sizes of the ICC on the minimum effect and power. The calculations were performed with the two-sample t-test, assuming the same variances in both arms.

The planning and implementation of the example study on the basis of this exploratory approach is pragmatic. This approach often appears to be more realistic than the confirmatory approach, because the latter often results in unfeasibly high sample sizes.

Confirmatory approach: Calculation of the sample size for a given power and minimum effect

The implementation of steps 1 to 5 of the schema shown in Figure 3 (Fig. 3) is described below for the example study. The outcome variable is assumed to be metric and approximately normally distributed. For the test arm, the score is assumed to change by a mean of 3 points (i.e. from 92.5 to 95.5, see above). The calculations are performed with the two-sample t-test, whereby the same variances are assumed in both arms (9.16, see above). For sample size calculation, a power of 80% and a type 1 error of 5% (two-sided) are assumed. First, this information is used to calculate the sample size for a study with individual randomization (e.g. [12]): the calculation shows that a total of 148 students per study arm (296 students in total) would have to be included in a study that randomizes individuals (i.e. does not consider the clustering). This number now has to be corrected by the design effect (DE=1.38, calculation see above): this correction indicates that 148·1.38~ 205 students would have to be included in each study arm (total study: 409 students, which would mean a total of approximately k=21 seminar groups). Table 4 (Tab. 4) shows the impact of the size of the ICC and seminar groups on the total sample size and the number of seminar groups for the above mentioned effects of the example study. The total sample size was rounded to the nearest whole number, and the number of seminar groups was rounded to the nearest even number because the example study will use a 1:1 randomization. This approach results in a higher actual total sample size than the total sample size given in the column N_tot_, which means that the power reaches values higher than 80%.

Because of the given framework conditions (maximum of 320 students, seminar group size m=20), it would not be possible to complete the study in one semester. However, it would not be advisable to perform the study over several semesters or as a multicenter study because of the considerable differences between different academic years (students and lecturers, other framework conditions) and universities. Consequently, a confirmatory study is not realistic in this setting. In such a situation, the exploratory approach therefore appears to be advisable, i.e. calculation of power or minimum effect for a fixed given sample size.

A modification of the design would be the use of a stratified cluster randomization by weekday (Tuesday, Thursday, Friday).

**Statistical analysis: **Because the study is rather small and has a small number of clusters and because the cluster size is almost constant, cluster-level analysis is recommended (see Section 4.5). One option to perform such an analysis would be to calculate cluster mean values from the study results and use the two-sample t-test. Further examples with practical examples of analyses of cluster-randomized studies are given in [30], [32], and [33].

## 6. Discussion and recommendations

In addition to studies with other designs (e.g. observational studies), prospective two-arm (intervention) studies are frequently used in educational research to compare different teaching methods. These studies should adhere to recognized standards and methods of scientific research, in particular the presence of a control arm and the achievement of statistical equivalence (i.e. structural equivalence – achieved by randomization and possibly stratification – and equivalence of treatment conditions and observations). Unless there is a legitimate reason not to do so, comparative scientific studies should no longer be performed without a control arm. Quasi-experimental studies with a control arm but without randomization should also be avoided. A major criticism of the results of such studies is the lack of structural equivalence combined with the risk of confounded effects. The best approach to avoid such problems is to assign the study participants to the study arms randomly, by either individual or cluster randomization. Because of the advantages of randomization, if at all possible the extra effort should be made, especially because the effort required to perform randomization is small compared with the effort associated with the entire study: the implementation of a study usually requires many resources, whereas randomization requires comparatively few. However, randomization results in large gains in the interpretability and validity of the study findings.

Compared with studies from other fields, however, studies in educational research have some special conditions and requirements that affect their planning, implementation, and analysis. Because of the existence of natural clusters, cluster randomization usually is the only option to perform randomization if there is a limited number of students and a given approximately constant cluster size. Furthermore, the time- and location-related availability of different resources, such as lecturers, seminar rooms, laboratories, lecture halls, and computer pools, must be considered. When calculating sample size, it is necessary to take the cluster structure into account because the outcome is more similar among students within a cluster (e.g. within seminar groups) than among students from different clusters (e.g. different seminar groups). Depending on the strength of this similarity (measured by the ICC), in cluster-randomized studies the sample size required to achieve a certain power can be significantly higher than the sample size of a corresponding study with individual randomization. For this reason, i.e. because of their limited maximum sample size, many studies in educational research have only exploratory character (for reasons of feasibility). Structural equivalence is particularly important so that differences found in the study can be attributed to the method being studied. When performing the statistical analysis of cluster-randomized studies, one should also ensure that an adequate statistical methodology is being used that gives appropriate consideration to the dependencies resulting from the cluster structure. Because of the complex statistical methods required in all phases of a cluster-randomized study, support from a competent expert with appropriate specialist knowledge is recommended during the practical implementation of such studies. Such experts may be scientific staff at biometric institutions, for example, which are part of most universities with a medical faculty.

In addition to the disadvantages mentioned in Table 1 (Tab. 1), compared with studies with individual randomization studies with cluster randomization have a higher risk of not achieving structural equivalence at the individual level, which may jeopardize the internal validity. Another reason to critically scrutinize internal validity is that cluster-randomized studies usually are not blinded [12]. Consequently, an adjustment for the unequally distributed characteristics must be made during the statistical analysis, e.g. by a suitable regression method [12], [13]. As with all clinical studies, even if cluster-randomized studies have internal validity external validity can only be established heuristically. This is probably more difficult in educational research studies than in clinical studies because the conditions at the various teaching institutions are so different. Because of the larger sample sizes and the more complex methodology, one should consider at the planning stage of educational research studies whether a cluster randomization is justified and necessary [34].

Finally, the following recommendations summarize important measures that take clusters into account and ensure the quality of prospective, two-arm studies in educational research:

1. Teaching is usually performed in groups of students, so that a natural cluster structure is given and cluster randomization is the most appropriate approach.
2. Attention must be paid to cluster randomization during study design, sample size calculation, analysis, and reporting.
3. A cluster-randomized study should not include too few clusters, i.e. no fewer than 8-10 [32].
4. In the case of very few or very different clusters, it may make sense to match clusters.
5. Blinding usually is not possible. The use of outcome variables that are as objective as possible and a blind assessment, for example the evaluation of PBL outcomes by independent people who are not participating in the study, is therefore recommended and serves to improve the internal validity.
6. As far as possible, structural equivalence should be maintained by creating the same conditions for the groups, for example the same times and seminar rooms for the study arms being compared.

On the basis of our experience and the arguments presented here, we recommend the use of control arms and suitable randomization in prospective, two-arm comparative educational research studies in order to achieve good and convincing results also in studies in this field. Cluster randomization can be a crucial building block in this context, and therefore it should be increasingly used in educational research studies.

## Acknowledgements

The authors thank Jacquie Klesing, Board-certified Editor in the Life Sciences (ELS), for editing assistance with the manuscript.

## Competing interests

The authors declare that they have no competing interests.

## Figures and Tables

**Table 1:**
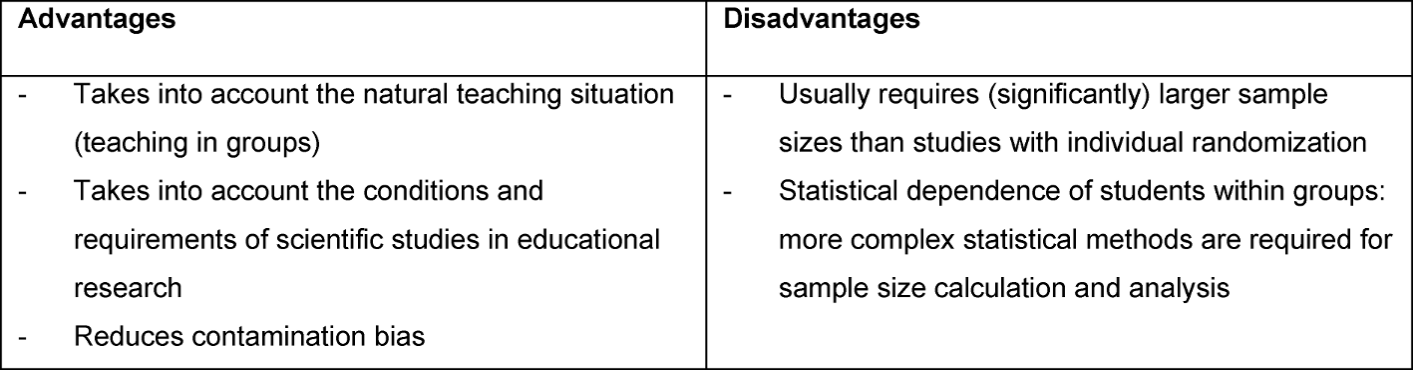
Advantages and disadvantages of cluster randomization in educational research studie (adopted from [15])

**Table 2:**
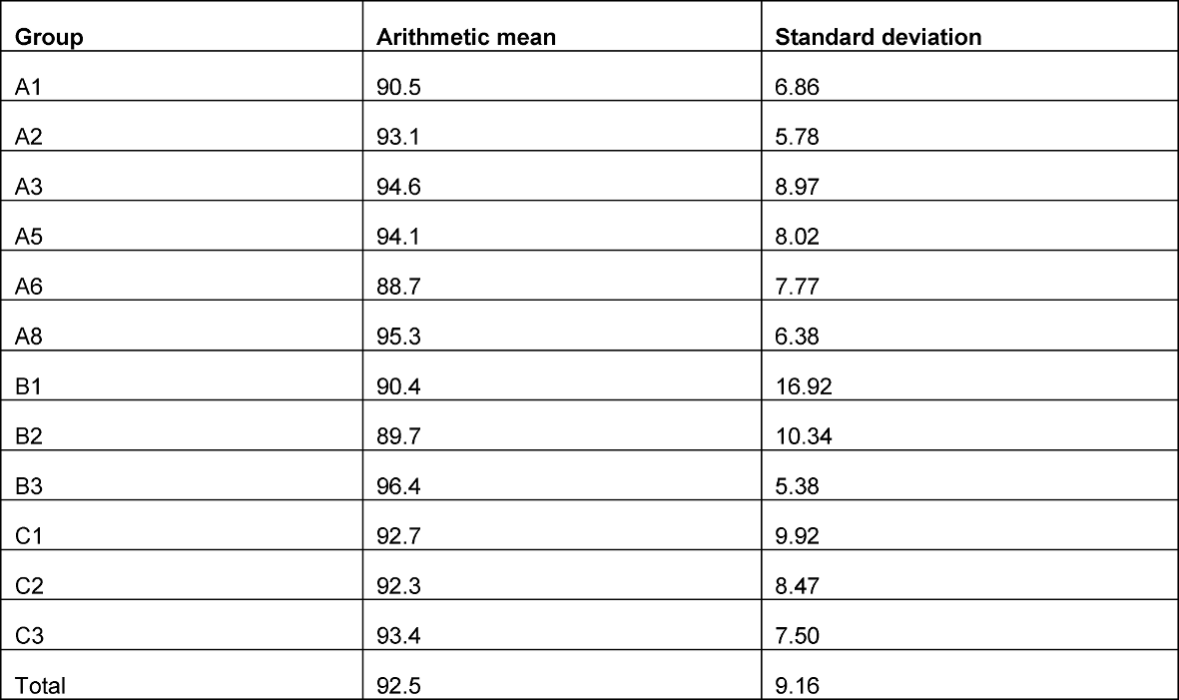
Results from the winter semester 2015/2016 cohort: arithmetic mean and standard deviations of the score in the total group and in the individual course groups

**Table 3:**
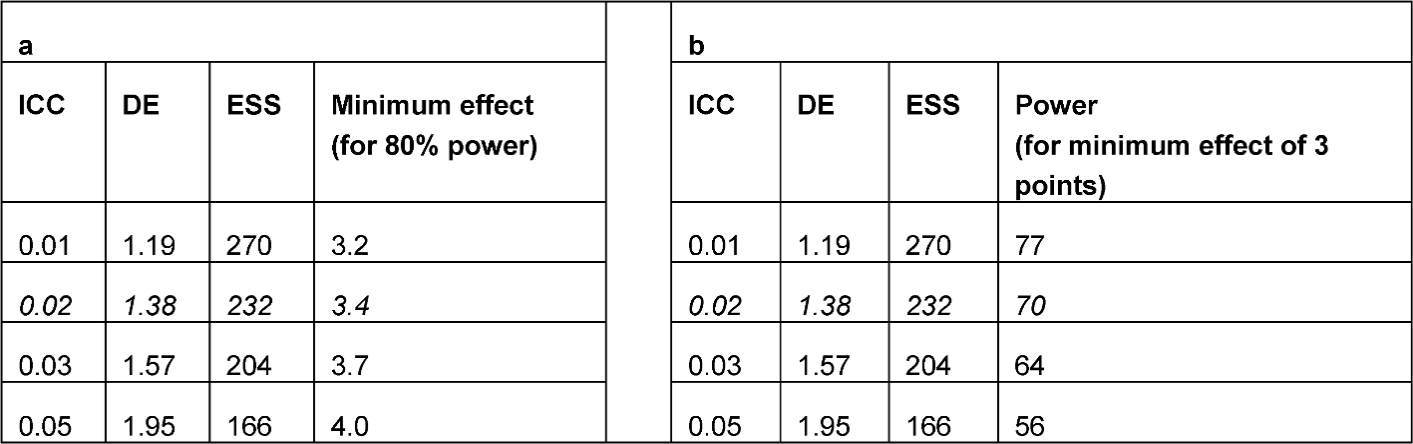
Impact of the size of the intracluster correlation coefficient (ICC) on the minimum effect (a) and power (b) for a predefined number of 320 students in 16 seminar groups with 20 students each. ESS = effective sample size (italics = study situation)

**Table 4:**
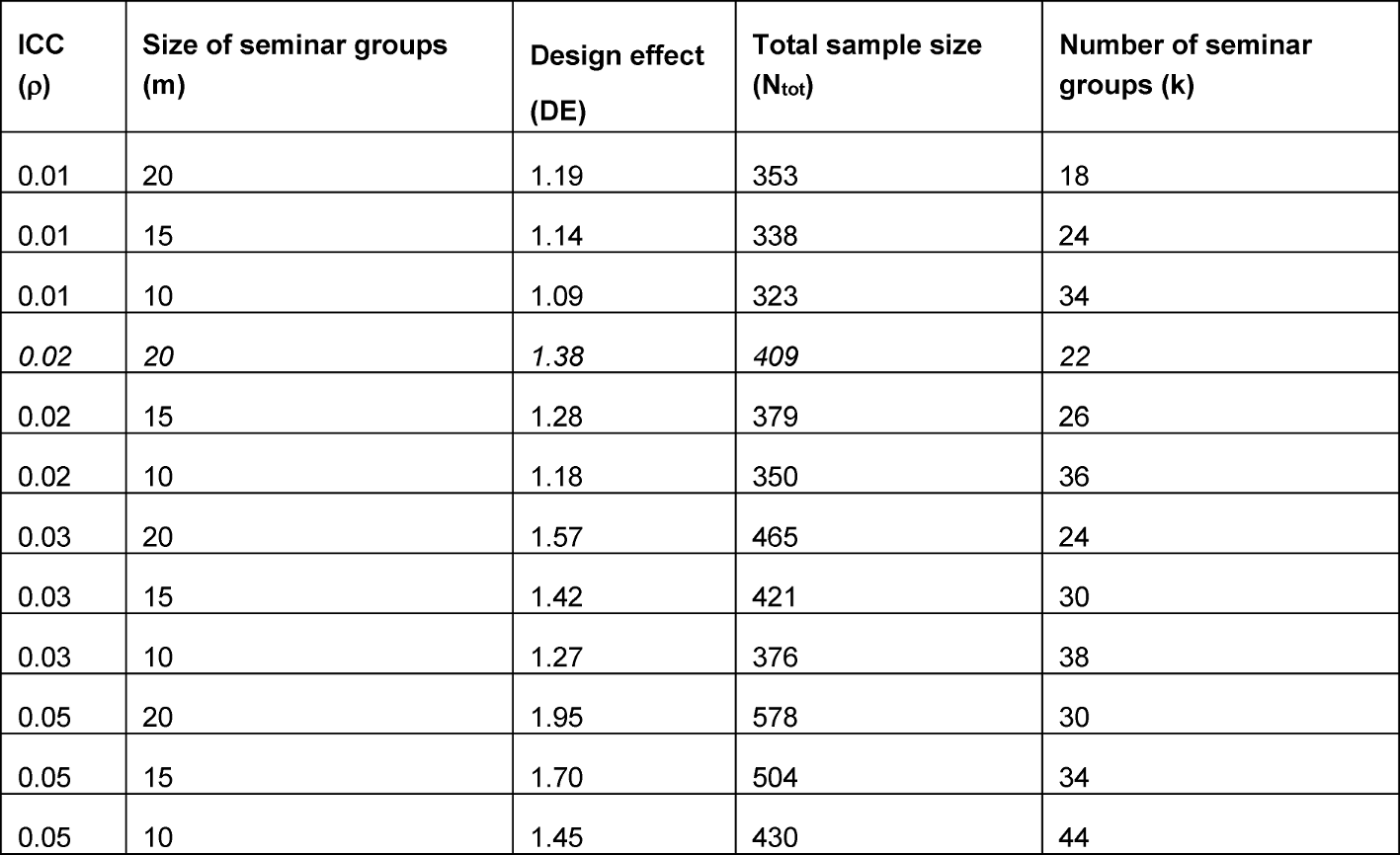
Impact of the size of the intracluster correlation coefficient (ICC) and the size of the seminar groups on the total sample size and the number of seminar groups in the overall study (italics = study situation)
